# Supplementary material for: Aspirin Foliar Spray-Induced Changes in Light Energy Use Efficiency, Chloroplast Ultrastructure, and ROS Generation in Tomato
Source: Int J Mol Sci. 2025 Feb 6;26(3):1368. doi: 10.3390/ijms26031368 (PMC11818874; doi:10.3390/ijms26031368)
Supplement: Supplementary file 1 [file ijms-26-01368-s001.zip › ijms-3427829-supplementary.pdf]

Article

Aspirin Foliar Spray-Induced Changes in Light Energy Use Efficiency, Chloroplast Ultrastructure, and ROS Generation in Tomato

Julietta Moustaka, Ilektra Sperdouli, Emmanuel Panteris, Ioannis-Dimosthenis S. Adamakis and Michael Moustakas

Table S1. Definitions of the chlorophyll fluorescence parameters used in the experiments

Parameter	Definition	Calculation
F_o	Minimum chlorophyll *a* fluorescence in the dark-adapted leaf (PSII centers open)	Obtained by applying measuring photon irradiance of 1.2 μmol photons m^{-2} s^{-1}
F_m	Maximum chlorophyll *a* fluorescence in the dark-adapted leaf (PSII centers closed)	Obtained with a saturating pulse (SP) of 6000 μmol photons m^{-2} s^{-1}
F_o'	Minimum chlorophyll *a* fluorescence in the light-adapted leaf	It was computed by the Imaging Win software V2.41a (Heinz Walz GmbH, Effeltrich, Germany) as $Fo' = Fo/(Fv/Fm + Fo/Fm')$
F_m'	Maximum chlorophyll *a* fluorescence in the light-adapted leaf	Measured with saturating pulses (SPs) every 20 s for 5 min after application of the actinic light (AL) of 426 μmol photons m^{-2} s^{-1} or 1000 μmol photons m^{-2} s^{-1}
F_s	Steady-state photosynthesis	Measured after 5 min illumination time before switching off the actinic light (AL) of 426 μmol photons m^{-2} s^{-1} or 1000 μmol photons m^{-2} s^{-1}
Φ_{PSII}	Effective quantum yield of PSII photochemistry	$(Fm' - Fs)/Fm'$
Φ_{NPQ}	Quantum yield of regulated non-photochemical energy loss in PSII	$Fs/Fm' - Fs/Fm$
Φ_{NO}	Quantum yield of non-regulated energy loss in PSII	Fs/Fm
Fv'/Fm'	Efficiency of the open PSII reaction centers	$(Fm' - Fo')/Fm'$
ETR	Electron transport rate	Φ_{PSII} × PAR × c × abs, where PAR is the photosynthetically active radiation, c is 0.5, and abs is the total light absorption of the leaf taken as 0.84
qp	Photochemical quenching, representing the redox state of quinone A (Q_A), or in other words the fraction of open PSII reaction centers based on the "puddle" model for the photosynthetic unit	$(Fm' - Fs)/(Fm' - Fo')$
NPQ	Non-photochemical quenching reflecting the dissipation of excitation energy as heat	$(Fm - Fm')/Fm'$
EXC	Excess excitation energy	$(1 - q_p)$ x Fv'/Fm'
1-qL	The fraction of closed PSII reaction centres based on the "lake" model for the photosynthetic unit	$1- (q_p \times F_o'/F_s)$
